# Immune signatures in human PBMCs of idiotypic vaccine for HCV-related lymphoproliferative disorders

**DOI:** 10.1186/1479-5876-8-18

**Published:** 2010-02-19

**Authors:** Luigi Buonaguro, Annacarmen Petrizzo, Marialina Tornesello, Maria Napolitano, Debora Martorelli, Giuseppe Castello, Gerardo Beneduce, Amalia De Renzo, Oreste Perrella, Luca Romagnoli, Vitor Sousa, Valli De Re, Riccardo Dolcetti, Franco M Buonaguro

**Affiliations:** 1 Lab. of Molecular Biology and Viral Oncogenesis & AIDS Reference Center, Istituto Nazionale Tumori "Fond. G. Pascale", Naples, Italy; 2 Lab of Clinical Immunology, Istituto Nazionale Tumori "Fond. G. Pascale", Naples, Italy; 3 Cancer Bio-Immunotherapy Unit, Centro di Riferimento Oncologico, I.R.C.C.S. - National Cancer Institute, Aviano, Italy; 4 Clinical Pathology, Istituto Nazionale Tumori "Fond. G. Pascale", Naples, Italy; 5 Haematology Unit, University of Naples "Federico II", School of Medicine, Naples, Italy; 6 VII Division of Infectious Diseases, Cotugno Hospital, Naples, Italy; 7 Areta International, Gerenzano, Italy; 8 Experimental and Clinical Pharmacology, Centro di Riferimento Oncologico, I.R.C.C.S. National Cancer Institute, Aviano, Italy; 9 Institute of Human Virology, University of Maryland School of Medicine, Baltimore, MD, USA

## Abstract

Hepatitis C virus (HCV) is one of the major risk factors for chronic hepatitis, which may progress to cirrhosis and hepatocellular carcinoma, as well as for type II mixed cryoglobulinemia (MC), which may further evolve into an overt B-cell non-Hodgkin's lymphoma (NHL).

It has been previously shown that B-cell receptor (BCR) repertoire, expressed by clonal B-cells involved in type II MC as well as in HCV-associated NHL, is constrained to a limited number of variable heavy (VH)- and light (VL)-chain genes. Among these, the VK3-20 light chain idiotype has been selected as a possible target for passive as well as active immunization strategy.

In the present study, we describe the results of a multiparametric analysis of the innate and early adaptive immune response after *ex vivo *stimulation of human immune cells with the VK3-20 protein. This objective has been pursued by implementing high-throughput technologies such as multiparameter flow cytometry and multiplex analysis of cytokines and chemokines.

## Introduction

Hepatitis C virus (HCV) is a Hepacivirus of the Flaviviridae family, mainly involved in hepatic disorders, including chronic hepatitis which may progress to cirrhosis in about 10-20% of cases and further to hepatocellular carcinoma in 1-5% of cirrhotic patients [1].

Subsequently, the virus has been implicated as one of the major risk factors for type II mixed cryoglobulinemia (MC), an autoimmune disease that may evolve into an overt B-cell non-Hodgkin's lymphoma (NHL) in about 10% of MC patients [2-5]. Several studies have contributed to establish the causative role of HCV infection in the etiopathogenesis of MC, showing the presence of the viral RNA and/or anti-HCV antibodies in a range of 70 to 100% of MC [6-8]. Furthermore, the clinical evolution of MC is closely linked to the natural history of the underlying HCV chronic infection [9,10].

The most accredited pathogenetic mechanism of MC during HCV chronic infection is the persistent immune stimulation sustained by viral proteins which, in turn, may result in production of cross-reactive autoantibodies, including cryoglobulins [11,12]. Chronic stimulation of the B-cell by HCV epitopes may produce the expansion of B-cell subpopulations with dominant genetic characteristics. In particular, the interaction between HCV E2 protein and CD81 molecule, an almost ubiquitous tetraspannin present on B-cell surface, has been shown and it may lead to a strong and sustained polyclonal stimulation of B-cell compartment [13]. Furthermore, the t (14,18) translocation observed in 85% of the patients affected by HCV-related type II MC might lead to abnormally elevated expression of Bcl-2 protein with consequent inhibition of apoptosis and increased B-cell survival [14]. This multistep process may ultimately lead to B-cell NHL as late complication of the MC syndrome [9,15].

The clonality of expanded B cells can be defined by the analysis of the antigen-binding region (so called idiotype, Id) of the immunoglobulin produced and expressed by the B-cell clone. According to the variety of Ids identified, the lymphoproliferative disorder may be sustained by mono-, oligo- or polyclonal B cells. It has been previously demonstrated that the B-cell receptor (BCR) repertoire expressed by clonal B-cells involved in HCV-associated type II MC as well as in NHL is not random, with V1-69, V3-7, V4- 59 variable heavy (VH)- and still more variable κ (VK)3-20 and VK3-15 light (VL)-chain genes being the most represented [16-18]. These data suggest a model of antigen-driven origin for these lymphoproliferative disorders with the recognition of a limited number of HCV antigens [18,19].

The constrained heterogeneity of Ids shared by such patients strongly suggests the possibility of targeting one or few idiotypes to hit and eliminate the B cell clone sustaining the HCV-associated NHL. One strategy is to generate idiotype-specific MAbs to be employed in a selective passive immunization [20]. An alternative strategy is to use an idiotype vaccine [21] in order to elicit an active humoral/cellular immune response as preventive and/or therapeutic approach against the expansion of the B cell clone sustaining the HCV-associated NHL.

We have previously shown that a multivariate and multiparametric analysis can predict the innate and early adaptive immune response induced by a vaccine molecule in human monocyte-derived dendritic cells (MDDCs) as well as whole peripheral blood mononuclear cells (PBMCs) using an ex-vivo experimental setting. This systems biology approach involves high-throughput technologies such as global gene expression profiling, multiplex analysis of cytokines and chemokines, and multiparameter flow cytometry, combined with computational modeling [22-26].

In the present study, we performed a multiparametric analysis of the innate and early adaptive immune response after *ex vivo *stimulation with the VK3-20 light chain protein, the idiotype most frequently identified on B cell clones sustaining the HCV-associated type II MC and NHL. This objective has been pursued using freshly isolated circulating human PBMCs.

## Materials and methods

### Enrolled subjects

Peripheral blood was obtained by venipuncture from 5 healthy volunteers and 10 HCV positive patients. All human specimens were obtained and processed at the National Cancer Institute in Naples under informed consent, as approved by the Institutional Review Board.

### Cell culture medium

PBMCs culture medium consisted of RPMI 1640 medium (Life Technologies, Carlsbad, CA) supplemented with 2 mM L-glutamine (Sigma), 10% fetal calf serum (Life Technologies) and 2% penicillin/streptomycin (5,000 I.U./5 mg per ml, MP Biomedicals).

MDDCs culture medium consisted of RPMI 1640 medium (Life Technologies, Carlsbad, CA) supplemented with 2 mM L-glutamine (Sigma), 1% non-essential amino acids (Life Technologies), 1% sodium pyruvate (Life Technologies), 50 μM 2-mercaptoethanol (Sigma) and 50 μg of gentamicin (Life Technologies) per ml.

### PBMC isolation and MDDC preparations

Fresh human PBMCs were isolated by Ficoll-Hypaque density gradient centrifugation and plated in six-well plates at a concentration of approximately 1 × 10^7 ^cells/well in a maximum volume of 3 ml/well for induction. Alternatively, MDDCs were generated as described previously [24,27], with minor modifications. Briefly, isolated PBMCs were enriched for CD14+ monocytes by negative selection with a cocktail of monoclonal antibodies (MAbs) from StemCell Technologies (Vancouver, British Columbia, Canada), according to the instructions of the manufacturer. Typically, greater than 80% of the cells were CD14+ after enrichment, as verified by flow cytometry. The isolated monocytes were allowed to adhere to plastic by plating in six-well plates at 1 × 10^6 ^cells per ml in RPMI 1640 medium for 2 hrs. Adherent monocytes were washed with RPMI 1640 medium and were then cultured for 6 days in DC culture medium supplemented with 50 ng of recombinant granulocyte-macrophage colony-stimulating factor (rGM-CSF; R&D Systems, Minneapolis, Minn.) per ml and 1,000 U of recombinant interleukin-4 (rIL-4; R&D Systems, Minneapolis, Minn.) per ml.

### Cell treatment

PBMCs or MDDCs were pulsed with serial dilutions of the recombinant VK3-20 protein (15, 5 and 1.5 μg/ml) provided by Areta International (Gerenzano, Italy) (Patent PCT/IB2008/001936). In parallel, cells were pulsed with 4 μg/ml of lipopolysaccharide (LPS), as positive control. PBS was used as negative control. After 16-h incubation, PBMCs and MDDCs were harvested and washed with 1× PBS (137 mM NaCl, 2.7 mM KCl, 10 mM Na_2_HPO_4_, 2 mM KH_2_PO_4_, pH 7.2) without Calcium and Magnesium.

### Flow cytometry

PBMCs and MDDCs were incubated for 30 min at 4°C with human monoclonal antibodies specific for CD40, CD80, CD83, CD86, HLA-DR, CD123, CD11c and CD14 (BD Pharmingen, San Diego, CA), washed and then analysed with a FACScalibur flow cytometer (BD Pharmingen). Data analysis was carried out with WinMDI2.8 Software.

### Multiplex cytokine analysis

At the time the cells were harvested, the supernatants were also collected and stored frozen until analyzed. Cytokine production was assessed using the BD™ Cytometric Bead Array (CBA) tool (Becton Dickinson and Company), according to the instructions of the manufacturer. Data acquisition was performed using a FACScalibur flow cytometer (BD Pharmingen), the analysis was performed with the BD CBA Analysis Software.

### Statistical analyses

Intergroup comparisons were performed with the Mann-Whitney U test (for univariate nonparametric group analysis). All p-values were two-tailed and considered significant if less than 0.05.

## Results

### Clinical parameters of subjects included in the analysis

Fifteen subjects were enrolled in the study. Ten subjects were HCV positive patients, of whom 2 were males and 8 were females (P1 - P10). Four of them were diagnosed with NHLs and only one of them showed a type II MC (Table 1). Five healthy subjects were enrolled as controls (C1 - C5), matched for age and life style.

**Table 1:** Clinical parameters of enrolled subjects

	SEX	HCV	MC	NHL
**C1**	M	Neg	Neg	Neg
**C2**	M	Neg	Neg	Neg
**C3**	F	Neg	Neg	Neg
**C4**	F	Neg	Neg	Neg
**C5**	F	Neg	Neg	Neg
**P1**	F	Pos	Neg	Neg
**P2**	M	Pos	Neg	Neg
**P3**	F	Pos	Neg	Neg
**P4**	M	Pos	Neg	Neg
**P5**	F	Pos	Neg	Neg
**P6**	F	Pos	n.d.	Follicular
**P7**	F	Pos	n.d.	Marginal
**P8**	F	Pos	n.d.	Diffuse large B cell
**P9**	F	Pos	Pos	Diffuse large B cell
**P10**	F	Pos	n.d.	Neg

n.d. = not done.

### VK3-20 protein induces comparable maturation phenotype in MDDCs and PBMCs of control subjects

**Figure 1:**
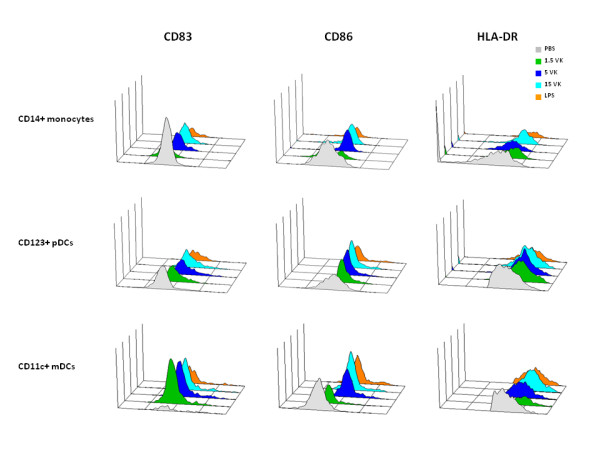
**PBMCs were incubated with increasing doses of VK3-20 protein for 16 hrs**. The expression of CD83, CD86 and HLADR was analysed by FACScalibur flow cytometer in CD14+ monocytes, CD123+ pDCs and CD11c+ mDCs. Data analysis was carried out with WinMDI2.8 Software. One representative experiment is shown.

**Figure 2:**
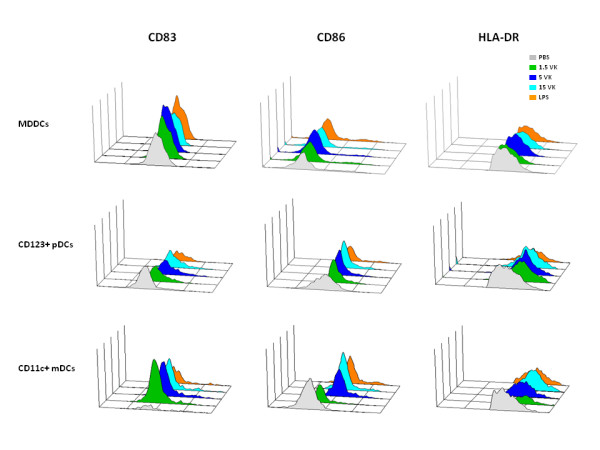
**Comparative analysis of the expression of surface maturation/activation markers (CD83, CD86, HLADR) performed on stimulated MDDCs, CD123+ pDCs and CD11c+ mDCs**.

Freshly derived PBMCs and immature MDDCs were obtained from healthy HCV-negative subjects and were incubated with 1.5 μg/ml, 5 μg/ml or 15 μg/ml of the VK3-20 protein. After a 16-hr stimulation, the expression of surface maturation/activation markers, such as CD40, CD80, CD83, CD86 and HLA-DR was examined. The results showed the up-regulation of all markers in PBMCs in CD14+ monocyte population as well as CD123+ plasmacytoid DC (pDC) or CD11c+ myeloid DC (mDC) (Fig. 1). Furthermore, MDDCs showed patterns of activation comparable to circulating mDCs and pDCs (Fig. 2).

**Figure 3:**
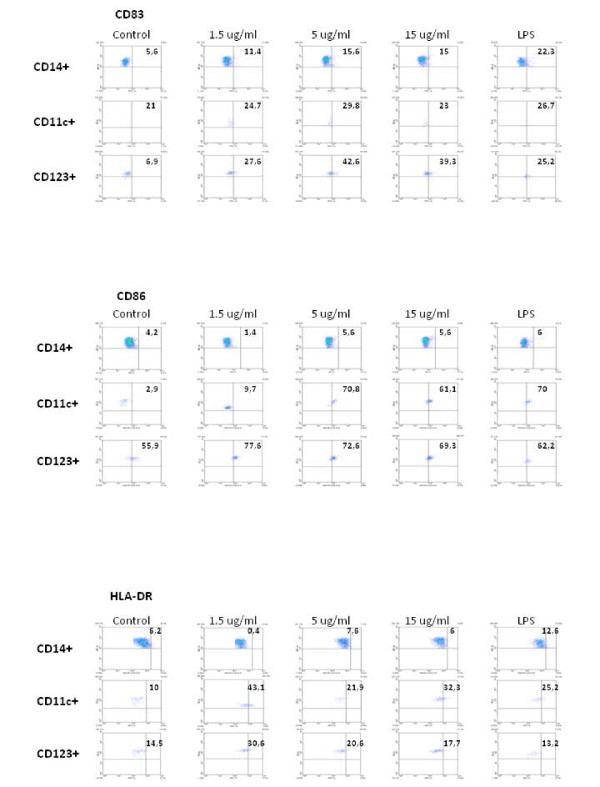
**6-color flow cytometric analysis was performed on VK3-20 protein-stimulated monocytes, mDC/pDC cell populations and immunophenotype analysis of surface maturation/activation marker expression is shown**. Values in each quadrant represent the percentage of positive cells.

Quantification of cells expressing activation markers in the subsets of circulating monocytes, pDC and mDC cells showed a trend of partial dose-response at increasing concentrations of the VK3-20 protein, indicating a specific activation/maturation activity on the circulating antigen presenting cells (APCs) (Fig. 3). The expression of CD40 and CD80 markers showed similar pattern of induction (data not shown).

The similar levels of activation/maturation observed in MDDCs and in PBMCs, regardless the marker of cell population used for gating, confirmed the feasibility of such analysis using "unselected" PBMCs, as previously reported [22,25].

### The VK3-20 protein induces maturation phenotype in PBMC

Given the comparable results observed in MDDC and in PBMC, subsequent analyses on samples from the enrolled subjects were performed only on circulating monocytes, pDC and mDC and the VK3-20-induced expression of the markers was evaluated in terms of mean fluorescence index (MFI).

**Figure 4:**
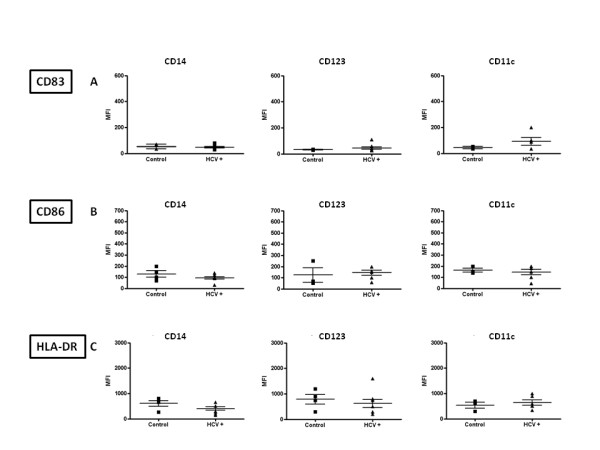
**Basal level expression of surface maturation/activation markers, indicated as Mean Fluorescence Index (MFI), on PBMC-derived monocytes and DC from control and HCV positive (HCV+) subjects**. CD14 = CD14+ monocytes; CD123 = CD123+ pDCs; CD11c = CD11c+ mDCs.

The basal expression of the markers was largely comparable between control and HCV+ subjects in the considered cell populations (Fig. 4A to 4C). The only exception is represented by basal CD83 expression, which shows a trend of higher expression in the CD11c+ mDC population of HCV+ subjects (Fig. 4A).

**Figure 5:**
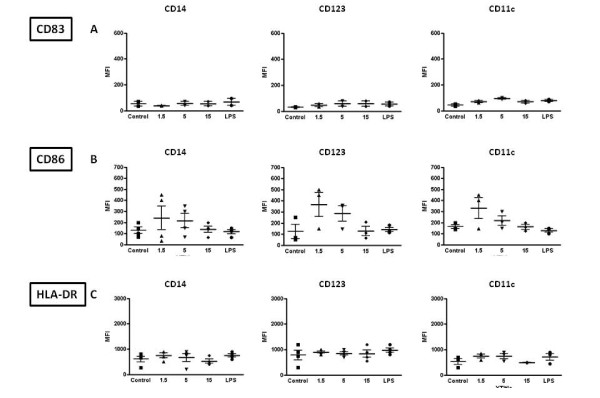
**Expression of surface maturation/activation markers, indicated as Mean Fluorescence Index (MFI), induced by the indicated concentrations of VK3-20 and LPS in PBMC-derived monocytes and DC from control subjects**. CD14 = CD14+ monocytes; CD123 = CD123+ pDC; CD11c = CD11c+ mDC.

**Figure 6:**
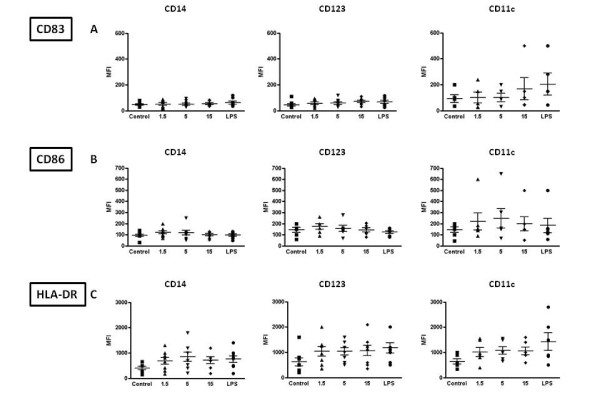
**Expression of surface maturation/activation markers, indicated as Mean Fluorescence Index (MFI), induced by the indicated concentrations of VK3-20 and LPS in PBMC-derived monocytes and DC from HCV seropositive subjects**. CD14 = CD14+ monocytes; CD123 = CD123+ pDC; CD11c = CD11c+ mDC.

The stimulation with VK3-20 protein induces a trend of increased expression of the activation/maturation markers in all circulating cells, from control and HCV seropositive subjects, although the most evident and consistent pattern is observed in the CD123+ pDC and/or CD11c+ mDC cells (Fig. 5 and 6A to 6C).

In particular, the lowest dose of VK3-20 used in the experimental system (1.5 μg) appears to be already sufficient to induce an increased expression of the activation markers in cells from both groups of subjects.

In control subjects, VK3-20 induced the most evident effect on the expression of CD86 in the circulating monocytes, pDCs and mDCs(Fig. 5B). On the contrary, the effect was significantly evident for all evaluated markers in the circulating cell populations from HCV + subjects (Fig. 6A to 6C). This observation suggests that overall the HCV seropositivity status does not significantly affect the responsiveness to an immunogenic stimulus (i.e., VK3-20) of circulating APC populations.

### Cytokine production in VK3-20-loaded PBMCs

In order to evaluate the impact of the VK3-20 protein stimulation on the production of cytokines involved in T-helper-cell activation, the levels of IL-2, gamma interferon (IFN-γ), tumor necrosis factor alpha (TNF-α), IL-6, IL-4 and IL-10 were assessed in the supernatant of PBMCs stimulated with the VK3-20 protein.

**Figure 7:**
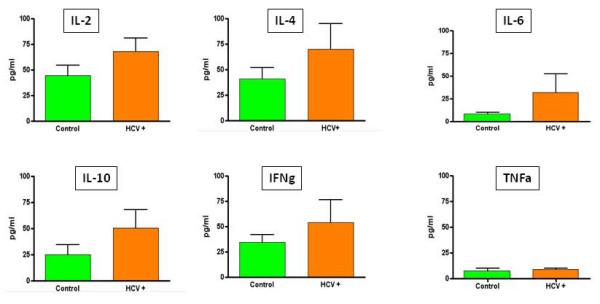
**Analysis of basal level production of Th1 and Th2 cytokines in supernatants of PBMCs from control and HCV positive (HCV+) subjects**.

**Figure 8:**
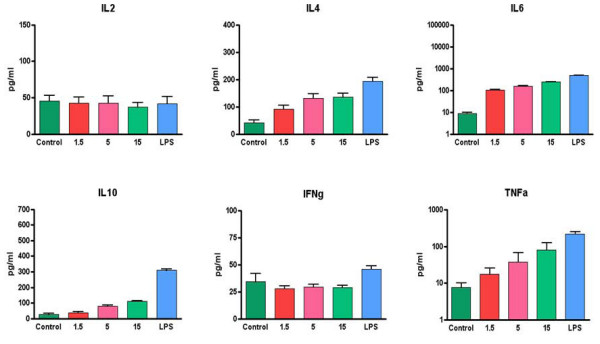
**Analysis of Th1 and Th2 cytokines in supernatants of PBMCs from control subjects induced by the indicated concentrations of VK3-20 and LPS**.

**Figure 9:**
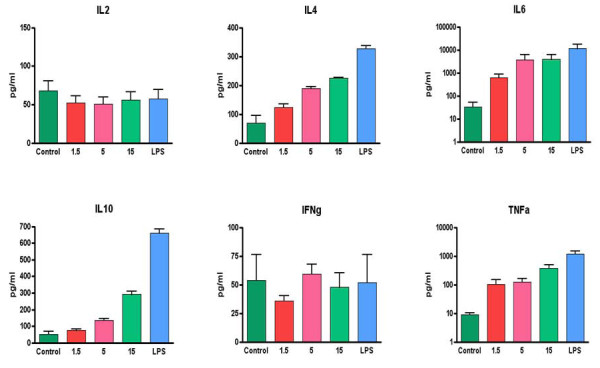
**Analysis of Th1 and Th2 cytokines in supernatants of PBMCs from HCV seropositive subjects induced by the indicated concentrations of VK3-20 and LPS**.

The average basal level of all evaluated cytokines showed no significant difference between HCV positive patients and control subjects (Fig. 7). Cell treatment with the VK3-20 protein did not induce any increase in the production of Th1 cytokines (IL-2 and IFN-γ). On the contrary, the VK3-20 protein induced a significantly higher production of the Th2 cytokines (IL-4, IL-6, IL-10, and TNF-α) in PBMCs from HCV seropositive and control subjects, with the highest levels observed in the samples treated with the highest concentration of VK3-20 (15 μg) (*p *< 0.05) (Fig. 8 and 9). The levels of Th2 cytokines induced in the HCV+ samples were significantly higher than those observed in control samples (*p *< 0.01).

## Discussion

The multivariate and multiparametric analysis described in the present study shows that the basal and VK3-20-induced expression of activation markers and co-stimulatory molecules in the evaluated circulating antigen presenting cells (APCs), CD14+ monocyte as well as CD123+ plasmacytoid DC (pDC) or CD11c+ myeloid DC (mDC) populations, is largely comparable between HCV-seropositive and control subjects. Overall, the markers show a trend of increased expression in all circulating cells, although the most evident and consistent pattern is observed in the CD123+ pDC and/or CD11c+ mDC cells. No significant difference was observed between results obtained in human monocyte-derived dendritic cells (MDDCs) and circulating APCs, confirming previous results from us and other groups [22,25,28,29].

The overall expression pattern suggests maturation/activation induced by VK3-20, although for some specific markers and in some patients the trend does not reach statistical significance. This observation suggests that the HCV seropositivity status does not significantly impair the immune activation status and the responsiveness of circulating APC populations to the VK3-20 immunogenic stimulus. Results obtained in parallel with lipopolysaccharide (LPS) used as a positive activation factor, confirm the responsiveness of circulating APCs from both groups analyzed in the present study. Nonetheless, some HCV+ individuals show a complete lack of maturation induced by VK3-20 in circulating APCs, strongly suggesting the need for individual evaluations to identify possible impairments in response to this immunogen.

The present results confirm and extend data from others showing a normal expression of surface molecules involved in antigen-specific T-cell activation on immature and mature DCs from HIV-1-infected and hepatitis C virus (HCV)-HIV-coinfected individuals [30-32]. Furthermore, monocyte-derived DCs from either HCV-infected or HCV-HIV-coinfected subjects have been previously shown to stimulate a mixed leukocyte reaction in purified, allogeneic CD4+ T cells comparable to that with DCs derived from healthy donors [33-35].

The average basal level of the Th2 (TNF-α, IL-6, IL-4, and IL-10) cytokines is significantly higher (*p *< 0.02) in HCV-seropositive compared to control subjects. On the contrary, Th1 cytokine levels are equivalent in the two groups. These results suggest a Th2 polarization induced by an established HCV infection, as previously extensively reported [36-39].

VK3-20 induced a significantly higher production of the analysed Th2 cytokines in PBMCs from HCV-seropositive and control subjects, with the highest levels observed in the samples treated with the highest concentration of VK3-20 (15 μg/ml) (*p *< 0.05). Furthermore, the levels of Th2 cytokines induced in the HCV+ samples were significantly higher than those identified in the control samples (*p *< 0.01), suggesting the persistence of a prevalent Th2 status. No increase in the production of Th1 cytokines (IL-2 and IFN-γ) was observed (*p *< 0.4) in the control as well as HCV+ group. In particular, the production of IFN-γ is known to be inhibited by IL-10 [40], with a sequential detrimental effect on the IL-12-mediated induction of IFN-γ production by NK and T cells [41-43]. Therefore, the high levels of IL-10 and TNF-α induced by VK3-20 could explain the lack of increased production of IFN-γ in both groups. The observed discrepancy between the VK3-20 concentration necessary for the maximal induction of activation markers (1.5 μg/ml) and the one necessary for the maximal induction of cytokine expression (15 μg/ml) may suggest a different pathway of activation involved in the two independent biological effects, which need further investigation.

The similar response observed in HCV-seropositive subjects, regardless of the diagnosis of type II MC or NHL, would suggest the absence of an *in vivo *priming for the VK3-20. In this regard, the expression of VK3-20 in the clonal B-cell populations of these subjects is currently under evaluation.

The impairment of basal and antigen-induced production of Th1-polarizing cytokines for HCV-seropositive individuals is in concordance with our previous observations on PBMCs from HIV infected subjects exposed *ex vivo *to a VLP-based HIV vaccine model [25,44].

The overall results here described represent a proof-of-concept and confirm the possibility of screening donor susceptibility to an antigen treatment using circulating APCs, CD14+ monocytes as well as CD123+ plasmacytoid DC (pDC) or CD11c+ myeloid DC (mDC) populations, without the need of purification and *ex vivo *selection of DCs, simplifying the identification of "responsive" vaccinees and providing mechanisms of eventual failures in individuals enrolled in clinical trials. When necessary, additional and more detailed studies on fractionated cell types would allow identification and a better characterization of the individual cells involved in mediating the *in vivo *response. In conclusion, our results indicate that circulating APCs from HCV-seropositive patients can be functional in active autologous immunotherapy strategies. In particular, the results strongly suggest the induction of the innate and early adaptive immune response by the protein corresponding to the VK3-20 light chain of the idiotype most frequently identified on B cell clones sustaining the HCV-associated type II MC and NHL. Therefore, its use as preventive as well as therapeutic vaccination strategy appears to be feasible and potentially effective. However, specific Th1-driving adjuvant strategies might be necessary to obtain the sought efficient therapeutic effect.

## Competing interests

MLN is the CEO of Areta International S.r.l., who provided the VK3-20 protein for the study. The authors declare that they have no competing interests.

## Authors' contributions

LB designed the study and wrote the paper; AP conducted the cellular inductions and cytokines evaluations; MLT conducted the statistical analyses; MN conducted the cytofluorimetric analyses; GC supervised the cytofluorimetric analyses; GB, AdR and OP provided the clinical samples; LR and VS provided the VK3-20 protein; DM, VdR participated to the design of experiment and evaluation of data; RD and FMB supervised the whole project.

All authors read and approved the final manuscript.
